# Localisation of AMPK γ subunits in cardiac and skeletal muscles

**DOI:** 10.1007/s10974-013-9359-4

**Published:** 2013-09-14

**Authors:** Katalin Pinter, Robert T. Grignani, Hugh Watkins, Charles Redwood

**Affiliations:** 1 Department of Cardiovascular Medicine, John Radcliffe Hospital, University of Oxford, West Wing Level 6, Oxford, OX3 9DU UK; 2 Present Address: Department of Paediatrics, Yong Loo Lin School of Medicine, National University of Singapore, Singapore, Singapore

**Keywords:** AMPK, γ Subunits, Sub-cellular localisation, Cardiomyopathy

## Abstract

The trimeric protein AMP-activated protein kinase (AMPK) is an important sensor of energetic status and cellular stress, and mutations in genes encoding two of the regulatory γ subunits cause inherited disorders of either cardiac or skeletal muscle. AMPKγ2 mutations cause hypertrophic cardiomyopathy with glycogen deposition and conduction abnormalities; mutations in AMPKγ3 result in increased skeletal muscle glycogen. In order to gain further insight into the roles of the different γ subunits in muscle and into possible disease mechanisms, we localised the γ2 and γ3 subunits, along with the more abundant γ1 subunit, by immunofluorescence in cardiomyocytes and skeletal muscle fibres. The predominant cardiac γ2 variant, γ2-3B, gave a striated pattern in cardiomyocytes, aligning with the Z-disk but with punctate staining similar to T-tubule (L-type Ca^2+^ channel) and sarcoplasmic reticulum (SERCA2) markers. In skeletal muscle fibres AMPKγ3 localises to the I band, presenting a uniform staining that flanks the Z-disk, also coinciding with the position of Ca^2+^ influx in these muscles. The localisation of γ2-3B- and γ3-containing AMPK suggests that these trimers may have similar functions in the different muscles. AMPK containing γ2-3B was detected in oxidative skeletal muscles which had low expression of γ3, confirming that these two regulatory subunits may be co-ordinately regulated in response to metabolic requirements. Compartmentalisation of AMPK complexes is most likely dependent on the regulatory γ subunit and this differential localisation may direct substrate selection and specify particular functional roles.

## Introduction

**Fig. 1:**
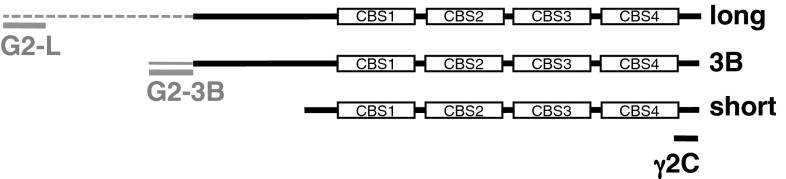
Mapping the γ2 immunogen sequences on the γ2 variants. Domain diagrams of the three γ2 variants showing the unique sequences of γ2-long and γ2-3B in *grey dotted* and *solid lines* respectively, along with the location of the immunogen sequences

AMPK regulates cellular energy homeostasis by monitoring the energy status of the cell (Hardie 2007). It is a hetero-trimeric complex (αβγ), α being the catalytic subunit. In mammals, there are two or three isoforms of each subunit (α1 and α2; β1 and β2; γ1, γ2 and γ3), each encoded by different genes (Hardie et al. 1998; Cheung et al. 2000). Differences in the tissue distribution of isoforms (Stapleton et al. 1997; Thornton et al. 1998; Turnley et al. 1999) and in muscle fibre type specific expression patterns have been reported (Durante et al. 2002; Winder et al. 2003; Mahlapuu et al. 2004). The regulatory γ subunits bind adenine nucleotides in the highly conserved nucleotide-binding domain consisting of four cystathionine-β-synthase (CBS) motifs. AMPK is allosterically activated when the AMP/ATP and ADP/ATP ratio increases; complexes containing the α2 and γ2 subunit isoforms are stimulated to a greater extent by AMP than those containing α1 and γ1 (Salt et al. 1998; Cheung et al. 2000; Oakhill et al. 2011), and those containing γ3 are least sensitive to AMP (Scott et al. 2004). The γ1 subunit is the most abundant and shows wide tissue expression, as does γ2 whereas the γ3 isoform is almost exclusively expressed in skeletal muscle (Lang et al. 2000; Barnes et al. 2004; Mahlapuu et al. 2004; Yu et al. 2004). Transcription from different promoters of the gene encoding AMPKγ2 (*PRKAG2*) generates at least three transcripts in cardiomyocytes (Fig. 1): one comprising the nucleotide binding domain only (γ2-short) and two longer transcripts (γ2-long and γ2-3B) both consisting of the nucleotide binding domain plus an N-terminal extension of different length (Cheung et al. 2000; Lang et al. 2000; Pinter et al. 2012a). The γ2-3B subunit is the predominant γ2 form in heart along with γ2-short, and its expression in other tissues is low or negligible; γ2-long is poorly expressed in the heart (Cheung et al. 2000; Lang et al. 2000; Pinter et al. 2012a).

Mutations in *PRKAG2* have been shown to cause cardiac hypertrophy with associated glycogen deposition, Wolff-Parkinson-White syndrome and conduction abnormalities (Blair et al. 2001; Gollob et al. 2001; Arad et al. 2002; Kim et al. 2009). All reported mutations are within the nucleotide-binding domain, and functional studies have suggested both that the basal level of activity is increased (Hamilton et al. 2001) and that nucleotide binding is lower or even abolished in the mutant protein resulting in impairment of AMPK activation (Steinberg and Kemp 2009). Interestingly, there is a reported mutation (R225Q) in γ3 occurring naturally in pig (Milan et al. 2000) and in human (R225W) (Costford et al. 2007); these mutations cause increased glycogen deposition in skeletal muscle in both cases, probably via increased glucose uptake rather than decreased glycogen utilisation (Andersson 2003). The amino acid affected by the γ3 mutation occupies the same position within the first CBS domain as the R302Q γ2 mutation.

The precise subcellular localisation of AMPK complexes within muscle cells is unclear. The β2 isoform, and hence trimers containing this subunit, was localized in the M-line in muscle fibres (Ponticos et al. 1998). In a different study, it was demonstrated that α1/γ1-containing AMPK is found in the Z-disk, apparently mediated by interaction of γ1 with plectin (Gregor et al. 2006), suggesting that, at least in this case, the regulatory subunit is responsible for AMPK compartmentalisation. In support of this, our work using human umbilical vein endothelial cells (HUVECs) also suggests that the γ subunit appears to determine AMPK localisation (Pinter et al. 2012b). Furthermore, selective activation of α2/β2/γ3 AMPK complexes during exercise has been reported in skeletal muscle and this was suggested to be due to the subcellular localisation of this AMPK complex, possibly directed by γ3 (Birk and Wojtaszewski 2006). Cell fractionation of mouse heart tissue found that all AMPK γ2 proteins were retained in the cytoskeletal fraction (Pinter et al. 2012a), suggesting a possible sarcomeric localisation. A yeast two-hybrid screen of a human heart cDNA library identified cardiac troponin I as an interactor with amino acids 1–273 of γ2-long, indicating that AMPK with γ2 is associated with the thin filaments (Oliveira et al. 2012).

As several different γ subunit isoforms and variants can and indeed are expressed in a cell (Cheung et al. 2000; Lang et al. 2000; Pinter et al. 2012a), we hypothesize that the different AMPK complexes have different functions, and function depends on their subcellular localisation that may be determined by the γ subunit. We have already demonstrated that AMPK complexes with distinct subunit compositions are compartmentalised and assigned for different cellular functions (Pinter et al. 2012b). In this study we provide further support for this notion by using immunofluorescence technique and detecting differential localisation of AMPK complexes with different γ subunits in mouse cardiomyocytes and in skeletal muscle fibres.

## Methods

### Animals and tissue collection

Ventricular cardiomyocytes were isolated from the heart C57BL/6 mice as described previously (Sears et al. 2003; Zhang et al. 2008); skeletal muscles (EDL, white quadriceps and soleus) were obtained from the same mouse strain.

### Immunofluorescent staining and confocal microscopy

Isolated mouse ventricular cardiomyocytes in cell suspension were spun onto poly-Lys-coated slides in a Statspin cytofuge (600 rpm, 2 min). Cells were fixed in 4 % PFA and permeabilized in 0.2 % Triton-PBS for 30 min. Blocking was carried out with 5 % BSA in PBS.

Bundles of skeletal muscle fibres were teased out in relaxing solution (10 mM EGTA, 5.6 mM MgCl_2_, 100 mM KCl, 20 mM imidazole, 5 mM ATP, pH 7.0; supplied with 10 mM creatine phosphate and 500 U/ml creatine kinase) onto poly-Lys-coated slides. Air-dried slides were rehydrated in PBS, then fixed and treated similarly to cardiomyocytes. Primary antibodies: rabbit anti-γ2 (γ2C—C-terminal), 1:60 dilution (gift from D. Carling); rabbit anti-γ2-3B (G2-3B), 1:50 (Pinter et al. 2012a); rabbit anti-γ2-long (G2-L), 1:50 [polyclonal antibody G2-L was raised in rabbit against the KHL-conjugated peptide 1-20 of γ2-long (MDTKKKKEVSSPGGSSGKKN-C) by Harlan UK (Hillcrest)]; rabbit AMPKγ3 (D-22) (Santa Cruz), 1:50 dilution; goat AMPKγ1 (T-20), 1:50 dilution (Santa Cruz); mouse anti-myomesin, 1:30 dilution (gift from E. Ehler); mouse monoclonal anti-α-actinin (EA53), 1:500 dilution (Sigma); goat L-type Ca^2+^ CP α1D (E-19), 1:50 dilution (Santa Cruz); goat SERCA2 (N-19), 1:50 dilution (Santa Cruz); mouse monoclonal anti-slow myosin heavy chain (BA-F8), 1:500 dilution (DSHB). Primary antibodies were usually applied overnight at 4 °C.

The appropriate fluorescent conjugated secondary antibodies (Alexa Fluor Molecular Probes) were used in 1:400 dilution; both the primary and the secondary antibodies were diluted in 5 % BSA/PBS. When it was possible, double staining was carried out. Cells were mounted using *SlowFade* ^*®*^Gold antifade reagent with DAPI (Invitrogen), however the nuclei were actually stained and imaged with To-Pro3/DNA (Invitrogen) and coloured in blue. Imaging was performed with a Leica TSC SP5 confocal laser-scanning microscope with a 63×, 1.4NA objective.

## Results

### Use of AMPKγ antibodies to localise AMPK complexes

AMPK is a trimeric complex and the subunits are not known to have any role as isolated proteins; therefore by detecting the γ subunits the subcellular localisation of the holoenzyme can be monitored. Differential localization of the γ2 protein variants is rather challenging. All γ2 variants share the entire sequence of γ2-short (Fig. 1) and therefore γ2-short cannot be selectively detected with antibodies. The available γ2 antibodies either recognise all three variants (if raised against sequence within the common nucleotide-binding region) or one (or both) of the longer forms (if raised against N-terminal sequences). The epitopes recognised by the three antibodies used in this study are mapped in Fig. 1. Antibody γ2C is a pan-γ2 antibody, with the immunogen being a short peptide at the C-terminus of all γ2 proteins; we have raised G2-L against the N-terminal peptide of 20 amino acids, and it exclusively detects γ2-long; and G2-3B, that only recognises γ2-3B (Pinter et al. 2012a).

### Localisation of AMPKγ isoforms in mouse cardiomyocytes

**Fig. 2:**
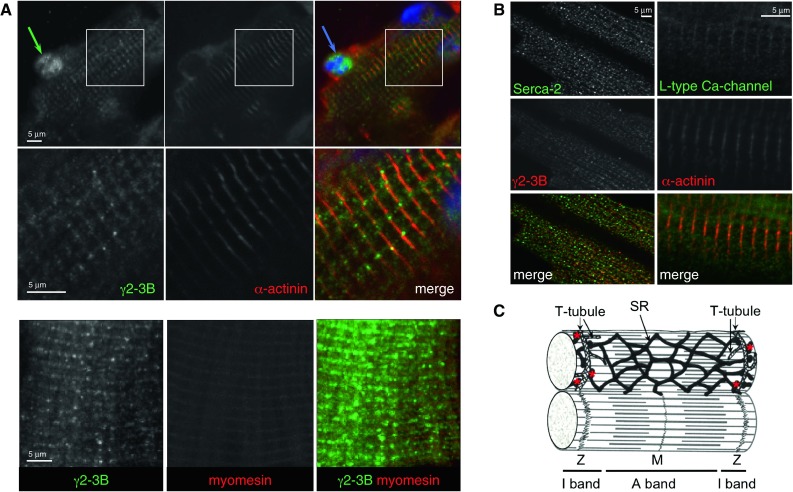
Subcellular localisation of AMPK γ2-3B in mouse ventricular cardiomyocytes. (**A**) Localisation of AMPK-γ2-3B—G2-3B antibody. Enlarged sections (*boxed areas*) are shown in the second row of images. (**B**) Staining pattern of the T-tubules and SR. Z-disk marker is α-actinin; M-line marker is myomesin. (**C**) Ultrastructure of cardiomyocytes—a schematic diagram showing the sarcomere and the T-tubule/SR system (based on Katz 1975); the terminal cisternae of SR are highlighted by the *red* stars

The G2-3B antibody that selectively reacts with γ2-3B, revealed a principally striated pattern with some nuclear staining. The striations aligned with the Z-disk as shown by co-staining with the α-actinin antibody (Fig. 2A). However, the staining is considerably more punctate and less uniform than that of α-actinin (higher magnification in the second row, Fig. 2A), suggesting that AMPK with γ2-3B may associate with structures that align with the Z-disk, such as the T-tubules and sarcoplasmic reticulum (SR). We tested this using antibodies against markers of T-tubules (L-type Ca^2+^ channel) and SR (SR Ca^2+^-ATPase, SERCA2); both of these antibodies produced punctate staining, more similar to γ2-3B than to the uniform α-actinin pattern (Fig. 2B). The terminal cisternae of the SR form irregular dyads in cardiomyocytes, hence the punctate staining along the Z-disk. In contrast to the T-tubules of skeletal muscle, those of the cardiomyocytes can run in a longitudinal as well as in a transverse direction (Soeller and Cannell 1999) and the punctate staining pattern with the T-tubule marker may arise from the longitudinal branches of the system. Schematic diagram of SR and T-tubular system in cardiomyocyte is shown in Fig. 2C.

**Fig. 3:**
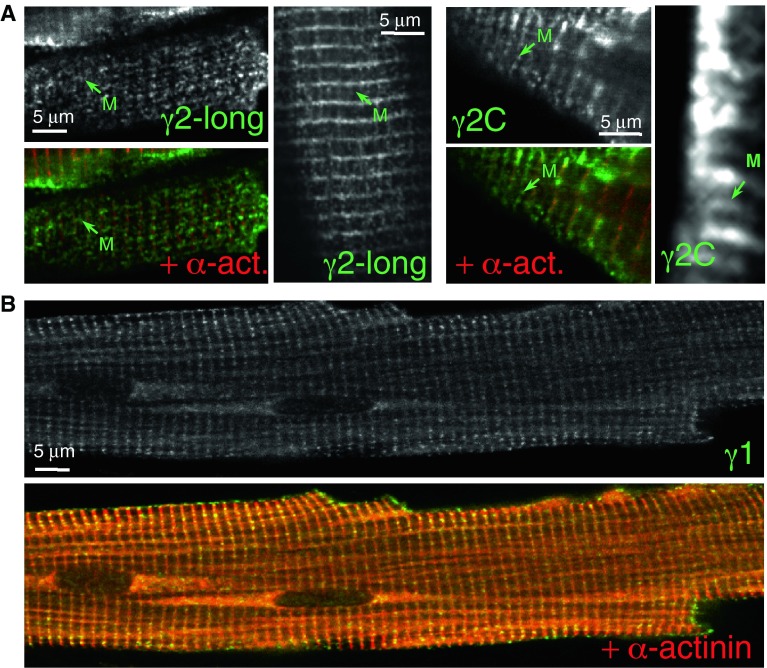
Subcellular localisation of γ2 and γ1 in mouse ventricular cardiomyocytes. (**A**) Staining patterns produced by antibodies to γ2-long (G2-L) and by a pan-γ2 antibody (γ2C). The strongest staining is around the Z-disk with these antibodies; antibodies G2-L and γ2C detect γ2 protein in the M-line. (**B**) Localisation of AMPK γ1 in the Z-disk by co-staining the cells with the Z-disk marker α-actinin antibody

The staining for the minority γ2-long variant produced a mainly Z-disk striated pattern, similar to the γ2-3B staining, along with some staining at the M-line (Fig. 3A). The C-terminal γ2C antibody, which detected three bands in Western blots of mouse heart tissue (Pinter et al. 2012a) produced clear staining of both the Z-disk and M-line regions (Fig. 3A). Whether the M-line signal reflects the presence of γ2-long variant (G2-L antibody detected some γ2-long protein in the M-line), or the γ2-short variant is unclear and is discussed later.

The γ1 protein was present in the Z-disk (Fig. 3B). This is consistent with the earlier report of its localisation in differentiated mouse myotubes, possibly recruited by binding plectin, a Z-disk component (Gregor et al. 2006). No nuclear staining was observed with the γ1 antibody in cardiomyocytes.

### Localisation of AMPKγ isoforms in mouse skeletal muscles

**Fig. 4:**
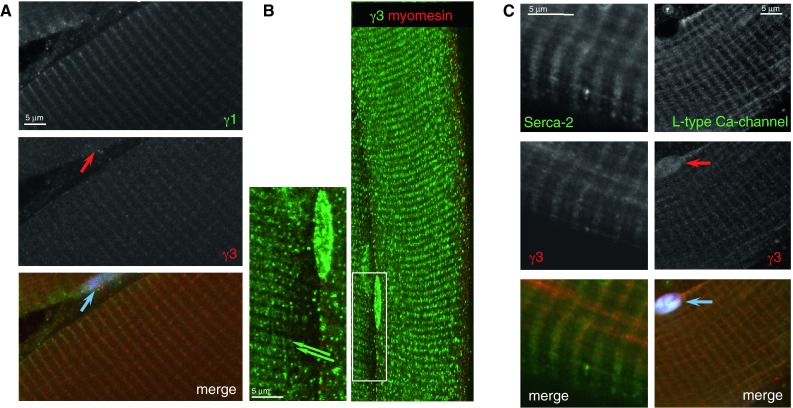
Immunofluorescence staining of mouse skeletal muscle fibres. (**A**) Localisation of the AMPK γ subunits in skeletal muscle fibres. AMPK with γ1 is in the Z-disk; the γ3 staining is more punctate around the Z-disk. There is γ3 staining in the nucleus. (**B**) The staining pattern for γ3 appears to be a very regular doublet flanking the Z-disk (enlargement of the boxed area, double green arrows). Nuclear staining with the γ3 antibody is very prominent. Marker for the Z-disk is γ1, and myomesin for the M-line. (**C**) Staining pattern of the T-tubules/SR system is similar to the γ3 staining. (**A,C**) EDL muscle fibres; (**B**) White quadriceps fibres

The γ3 regulatory subunit is mainly expressed in white, glycolytic fibres of adult skeletal muscle (Mahlapuu et al. 2004). The majority of fibres is type IIB in white quadriceps muscle of mouse (~94 %; fast, glycolytic fibres) and large proportion of fibres in EDL are also glycolytic (Bloemberg and Quadrilatero 2012). We isolated and stained bundles of fibres from EDL and from white quadriceps muscles and found γ3 staining around the Z-disk (Fig. 4A), but it is broader than the γ1 staining and some γ3 staining is also detectable in the nucleus; nuclear staining is more obvious in Fig. 4B. The γ3 staining appears as a highly uniform doublet along the Z-disk (Fig. 4B); the depicted, well-organised structure can be seen in the enlarged segment of the image.

**Fig. 5:**
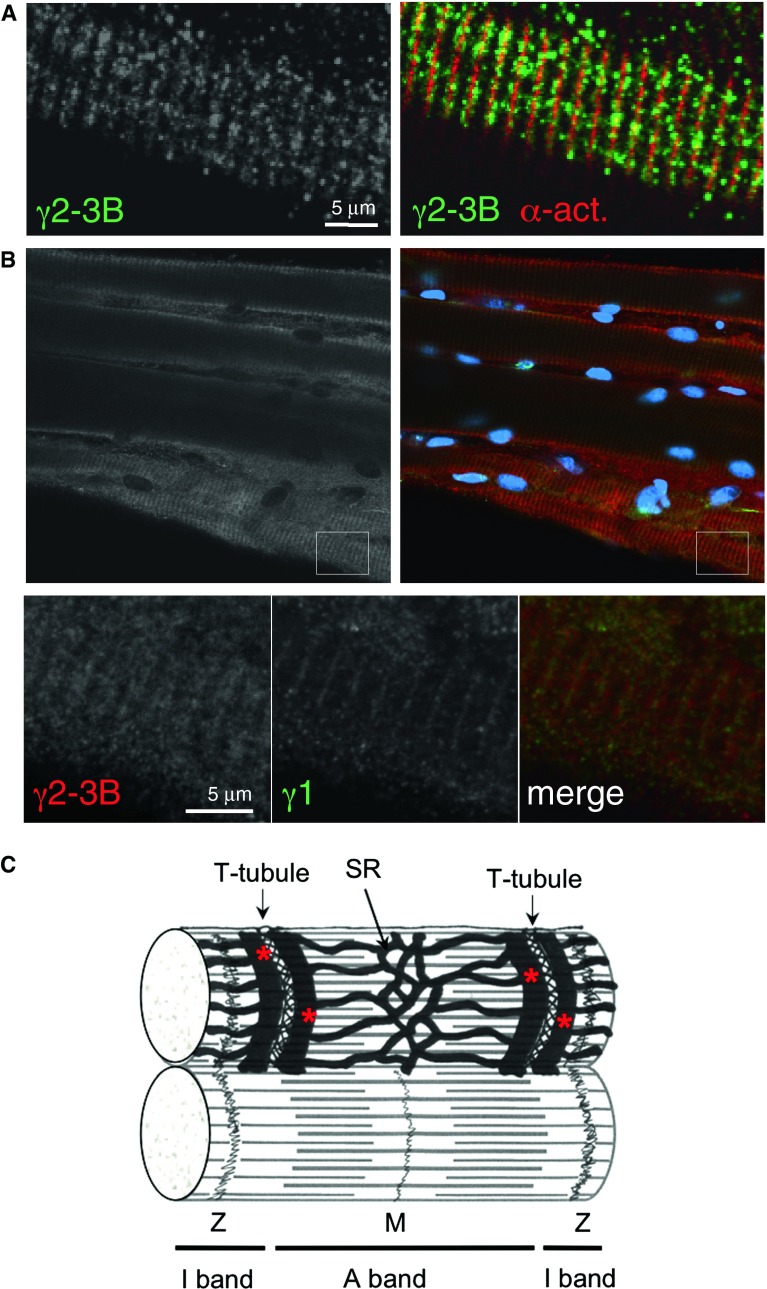
Detection of γ2-3B in skeletal muscle fibres. (**A**) EDL fibre stained with G2-3B and α-actinin antibodies. (**B**) Soleus fibres; the Z-disk marked by γ1 staining. The boxed area is enlarged and showing the regular γ-3B staining pattern that is around the Z-disk (*second row* of images). (**C**) Ultrastructure of skeletal muscle fibre—a schematic diagram (based on Eisenberg et al. 1974); the terminal cisternae of SR are highlighted by the *red* stars

The T-tubule marker (L-type Ca^2+^ channel antibody) and the SERCA2 antibody decorate the T-tubule/SR system in skeletal muscle fibres (Fig. 5C). Since the terminal cisternae of the SR form triads with the T-tubule in skeletal muscle, the pattern is very regular, rather similar to staining pattern shown for γ3 (Fig. 5B).

We have previously detected γ3 expression in the developing mouse heart; this declines after birth and appears to be “replaced” by γ2-3B expression (Pinter et al. 2012a). The γ subunit “switch” seems to coincide with metabolic changes: the embryonic heart is mainly glycolytic while the adult heart is oxidative. We therefore tested whether the oxidative skeletal muscle fibres contained AMPK γ2-3B. When EDL or white quadriceps fibres were stained with the γ2-3B antibody, we detected, though infrequently, fibres that were stained with γ2-3B antibody; the staining pattern was similar to the γ3 staining (Fig. 5A). In contrast, the occurrence of γ2-3B-stained fibres in soleus muscle was higher and imaging is shown in Fig. 5B. The pattern of γ2-3B staining resembles that of γ3 (see enlarged segment, Fig. 5B), suggesting that the two subunits occupy similar positions. No γ2-3B staining was detected in the nuclei (Fig. 5A). Interestingly, skeletal myopathy has been observed in patients with *PRKAG2* mutations (Murphy et al. 2005); ragged red fibres with excess mitochondria were detected but skeletal muscle biopsies of patients presented little glycogen accumulation.

**Fig. 6:**
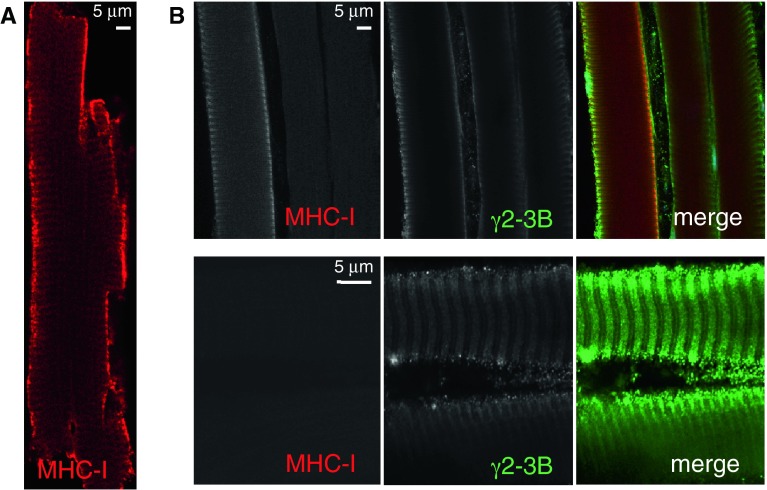
Expression of γ2-3B and slow myosin heavy chain in cardiac and skeletal muscle. (**A**) Cardiomyocyte stained with anti-slow myosin heavy chain (MHC-I); (**B**) A selection of soleus muscle fibres stained with both G2-3B and MHC-I antibodies. The weak M-line staining with G2-3B may depict the M-line part of the SR (see diagram in Fig. 5C)

Although a large proportion of fibres are slow oxidative type I in mouse soleus muscle (~30 %), about 50 % of the population are fast oxidative type IIA fibres (Bloemberg and Quadrilatero 2012). As type I fibres can be identified by their β myosin heavy chain content, therefore we performed double staining with a slow myosin heavy chain antibody (MHC-I) and with G2-3B antibody. The monoclonal MHC-I antibody recognizes both α and β myosin heavy chains and decorated cardiomyocytes as expected (Fig. 6A). Some soleus fibres react with both the MHC-I and with the γ2-3B antibodies but not all γ2-3B-reactive fibres are co-stained with MHC-I (Fig. 6B). The fibres that are not stained by the slow myosin antibody most likely are the fast oxidative type II fibres.

## Discussion

In mouse cardiomyocytes, the predominant AMPK γ2 protein, γ2-3B, was shown to give patchy Z-disk staining and was also found in the nuclei. The punctate Z-disk staining was similar to the T-tubule and SR markers, L-type Ca^2+^ channel and SERCA2 respectively. Staining with a pan-γ2 antibody suggested additional M-line localisation of either the γ2-short or γ2-long variants; the latter was confirmed with a γ2-long-specific antibody. Since the affinity of the γ2C antibody is the same for each γ2 protein, the higher staining intensity in the Z-disk may reflect the presence of the γ2-short, which is the second most abundant γ2 protein in cardiomyocytes after γ2-3B (Pinter et al. 2012a). The AMPK γ2 variants thus appear to localize to different sarcomeric positions in cardiomyocytes.

Our earlier work has shown that AMPK can phosphorylate cardiac troponin I, both in vitro and in vivo, and that this phosphorylation modulates the myofilament Ca^2+^-sensitivity (Oliveira et al. 2012). This finding was initiated by the identification of the interaction of cardiac troponin I with a fragment containing the N-terminal 273 amino acids of AMPK γ2-long by Yeast-Two-Hybrid screening of a human cardiac cDNA library. However, γ2-long and γ2-3B have a common segment in their N-terminal extension, and since there is much more γ2-3B protein than γ2-long in cardiomyocytes (Pinter et al. 2012a), that function may be linked to AMPK containing γ2-3B. Given that there is no apparent staining throughout the I band, it is possible that AMPK is recruited to phosphorylate cardiac troponin I and mobilised from its position around the Z-disk upon activation.

The γ1 protein was localised at the Z-disk in cardiomyocytes. AMPK with the γ1 isoform is known to be anchored by plectin to the Z-disk in differentiated myocytes (Gregor et al. 2006) and our observation is consistent with this. The γ2-short, similarly to γ1, only contains the nucleotide-binding domain and the highly conserved segment close to its N-terminus to where the β subunit binding-site was localised (Viana et al. 2007). We have reported that γ2-short is largely replaced by γ1 during cardiogenesis (Pinter et al. 2012a), suggesting partially or entirely overlapping function for the two proteins and that may support the Z-disk localisation of γ2-short. Interestingly, the Z-disk staining with the γ1 antibody resembles the subcellular positions of glycogen synthase (GS) that is regulated by AMPK (Prats et al. 2005; Bendayan et al. 2009); glycogen particles are also linked to the cytoskeleton and so are the glycogen-metabolising enzymes (Gregor et al. 2006; Graham et al. 2010). Therefore it is plausible to presume that AMPK with γ1 is involved in the regulation of glycogen metabolism along with AMPK containing the γ2-short subunit.

Both γ2-3B and γ3, in cardiac and skeletal muscle respectively, are present along the Z-disk and in the I band; both staining patterns closely resemble the respective T-tubule/SR structures. The different appearance of γ2-3B and γ3 staining reflects the structural differences of the T-tubules/SR structures in cardiac and skeletal muscle. In cardiomyocytes, the T-tubules are aligned with the Z-disk but a set of two T-tubules, flanking the Z-disk is present in the I band of skeletal muscle sarcomeres. In cardiac muscle, the arrangement of the terminal cisternae of SR is not as regular as in skeletal muscle, therefore diads flanking the Z-disk are formed instead of triads. The structure of the T-tubule system is also complex, with irregular branching in heart (Soeller and Cannell 1999).

Given their apparently common T-tubule/SR localisation, is there any indication that AMPK with γ2-3B or γ3 have similar functions? A point mutation in γ3 causes glycogen accumulation in skeletal muscle; the cause of this is not the activation of glycogen synthase or decreased glycogen utilization but increased glucose uptake (Andersson 2003). This mutation is in the first CBS domain, in the exact position as one of the γ2 mutations is; the consequence of the γ2 mutation is glycogen deposition in cardiac tissue (Gollob et al. 2001; Gollob 2003). The embryonic heart is more glycolytic but becomes more oxidative during differentiation; in parallel, there is an apparent switch from γ3 to γ2-3B expression in the developing heart (Pinter et al. 2012a). Glucose transport is mainly mediated by GLUT1 and GLUT4 in cardiomyocytes (Stanley et al. 1997) and myocardial AMPK activation and subsequent GLUT-4 translocation to the sarcolemma was reported in rat (Russell et al. 1999). The sodium/glucose cotransporter (SGLT1) is also expressed in heart (Banerjee et al. 2009) and AMPK activation was also reported to trigger the increased membrane translocation of SGLT1 (Sopjani et al. 2010).

In skeletal muscle, α2/β2/γ3 complexes become preferentially activated during exercise (Birk and Wojtaszewski 2006); α2-AMPK was identified as an endoplasmic reticulum (ER) stress suppressor as its activation maintains SERCA activity and intracellular Ca^2+^ homeostasis (Dong et al. 2010). The expression γ3 is restricted to glycolytic fast fibres; oxidative fibres (slow or fast) seem to contain γ2-3B. Mitochondria are tethered to the SR in both skeletal muscle fibres and in cardiomyocytes (Boncompagni et al. 2009) where we detected γ2-3B. It has been reported that AMPK phosphorylates PGC1α and instead of affecting the mitochondrial oxidative capacity it stimulate mitochondrial biogenesis (Jager et al. 2007). A *PRKAG2* mutation was shown to cause myopathy in red muscle (Murphy et al. 2005) and ragged red fibres were observed with mitochondria accumulation but without substantial glycogen deposition. This observation also supports our finding that γ2-3B is only expressed in oxidative muscle (cardiomyocytes, slow type I and fast type II skeletal muscle fibres), however we have not done the thorough fibre type matching.

Furthermore, several ion channels are regulated by AMPK, some of them by direct phosphorylation (reviewed by Andersen and Rasmussen 2012). In a recent study AMPK phosphorylation of the voltage-gated Kv2.1K-channel was demonstrated in neurons, where AMPK activation reduced excitability to conserve energy (Ikematsu et al. 2011). Kv2.1 is present in all part of the transverse and axial tubule system in cardiomyocytes (O’Connell et al. 2008). Ion channel down-regulation by AMPK has been reported; one of them is the KCNQ1 potassium channel. KCNQ1 ubiqutination is promoted by AMPK activation via the ubiqutin-protein ligase, Nedd4-2 in kidney cells (Alzamora et al. 2010). KCNQ1 is expressed in cardiomyocytes, where its abnormal trafficking was linked to hereditary long QT syndrome (Wilson et al. 2005). AMPK associated with the T-tubules may regulate ion transport, contributing perhaps to conductive irregularities that accompany cardiac hypertrophy caused by *PRKAG2* mutations.

In summary, we have demonstrated AMPK compartmentalisation in cardiomyocytes and in skeletal muscle fibres and showed that differential localisation of the different AMPK complexes is most likely governed by the regulatory subunits, either by the different γ isoforms or by the variants of γ2. By compartmentalisation, the different AMPK complexes are most probably assigned different functions. Mutation in the γ2 proteins would alter these functions; as a consequence, this could trigger mechanisms to cause the different aspects of the cardiac disease (hypertrophy, conductive disorder, glycogen deposition). However, further studies needed to understand the importance and the dynamics of the compartmentalisation of the γ2-AMPK complexes, focusing on specific interactions in each location. The need for isoform-selective activation of AMPK to develop cardioprotective therapies has been highlighted in a recent review article (Kim and Tian 2011) and protein–protein interaction studies could result in new drug designs that acts specifically on γ2-AMPK complexes to ameliorate the disease caused by the *PRKAG2* mutations.
